# Supplementary figures and images for: The phytochemical diversity of commercial Cannabis in the United States
Source: PLoS One. 2022 May 19;17(5):e0267498. doi: 10.1371/journal.pone.0267498 (PMC9119530; doi:10.1371/journal.pone.0267498)

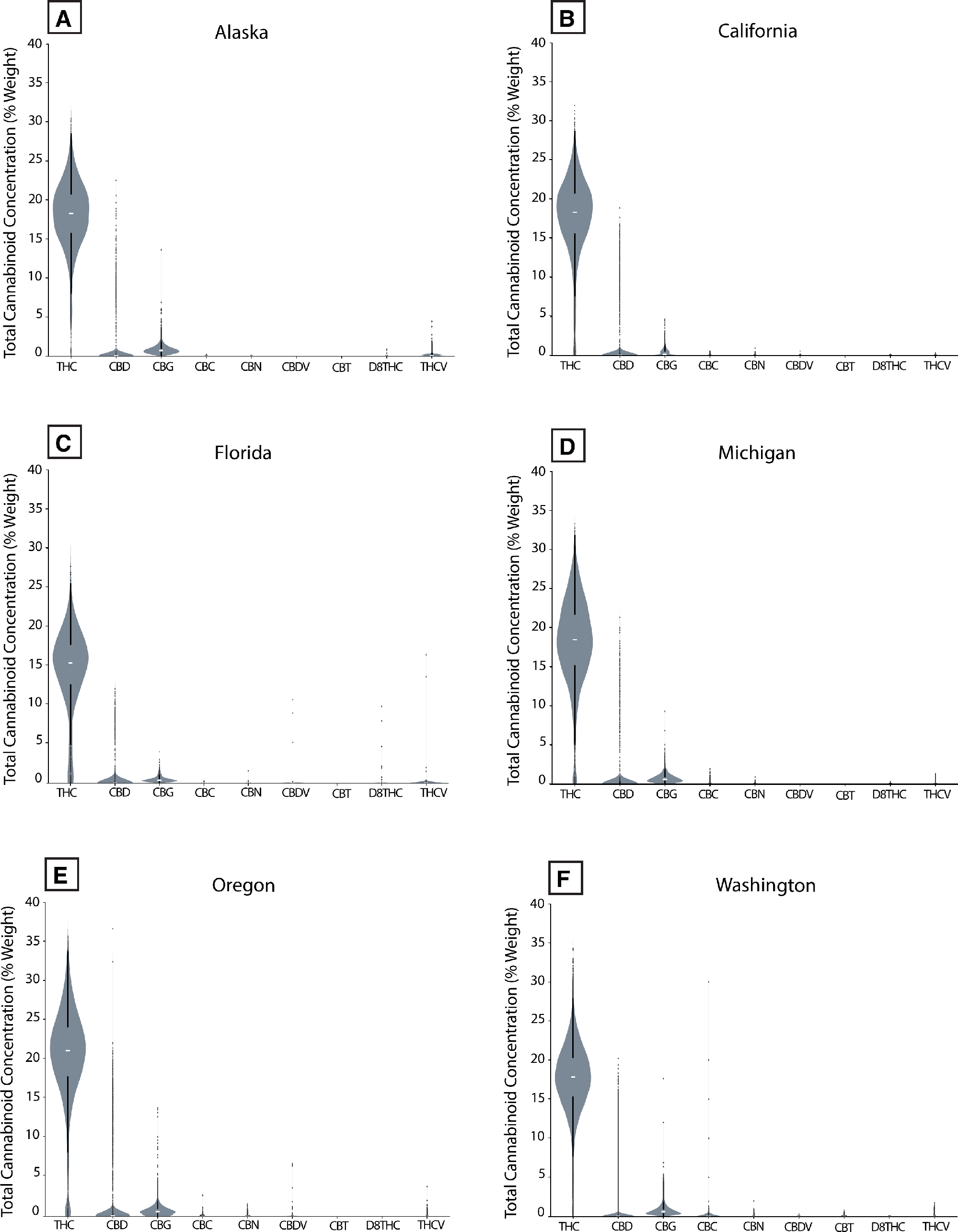

Supplement: S1 Fig — (TIF) [file pone.0267498.s001.tif]

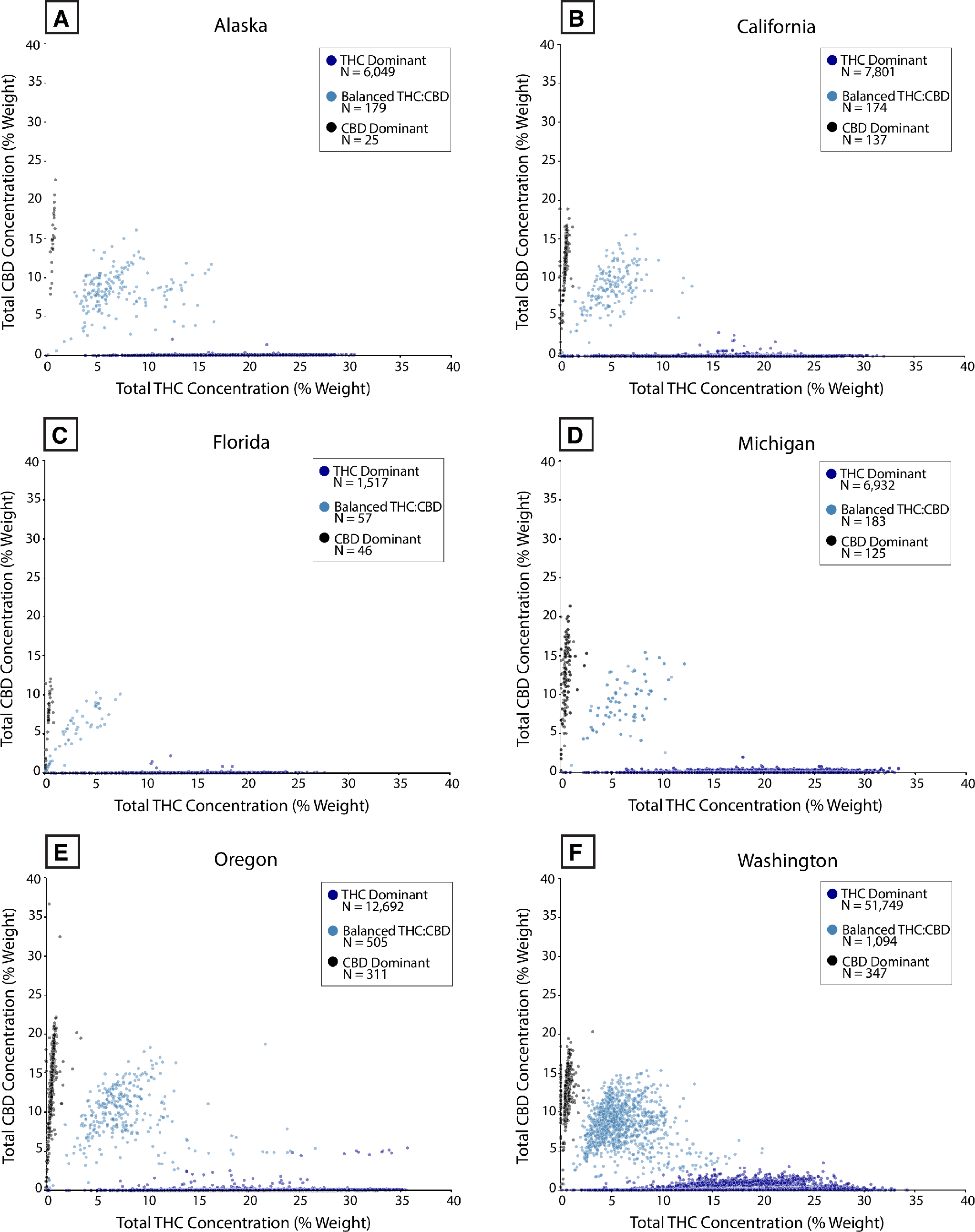

Supplement: S2 Fig — (TIF) [file pone.0267498.s002.tif]

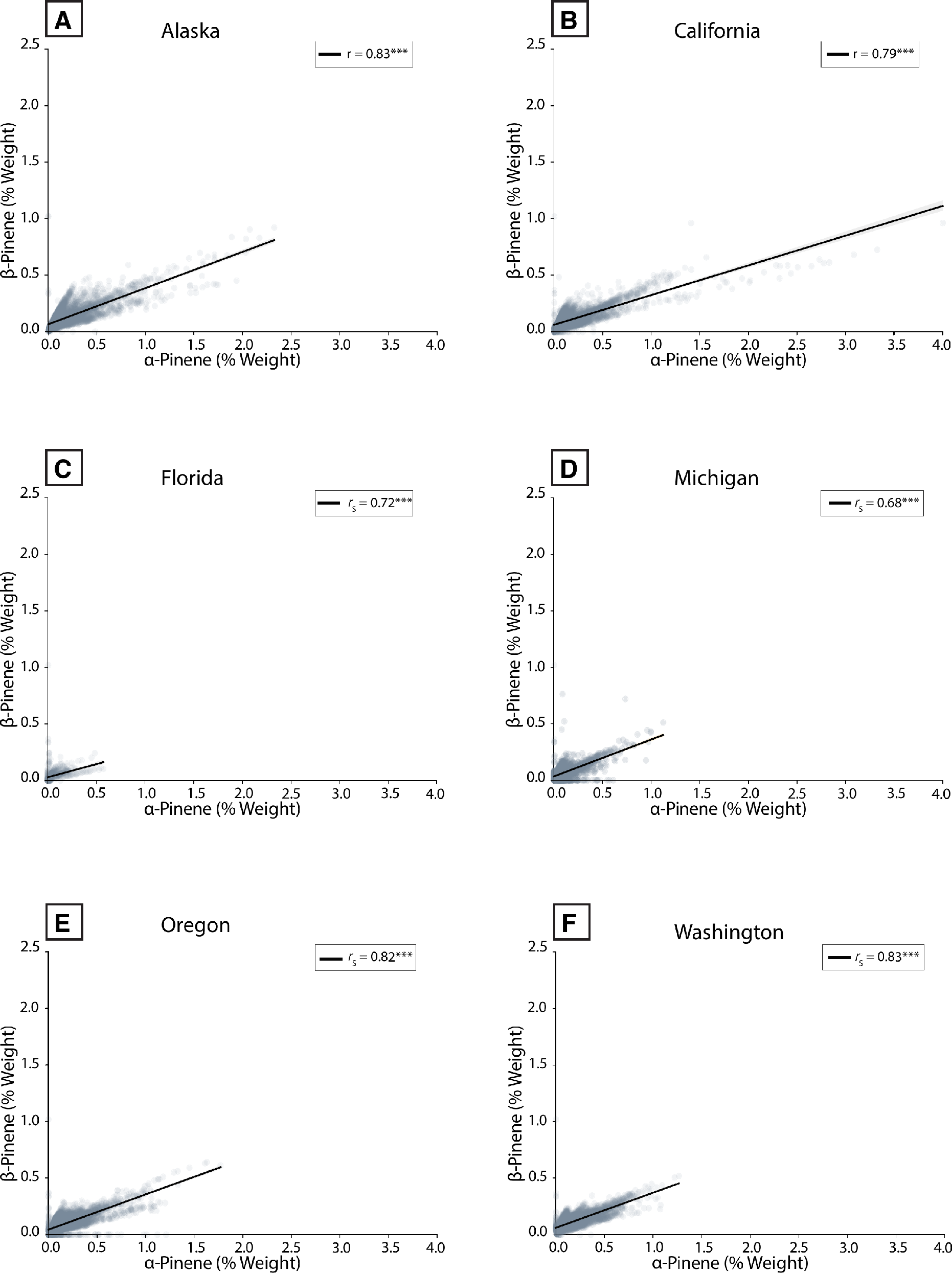

Supplement: S3 Fig — ***P < 0.0001. (TIF) [file pone.0267498.s003.tif]

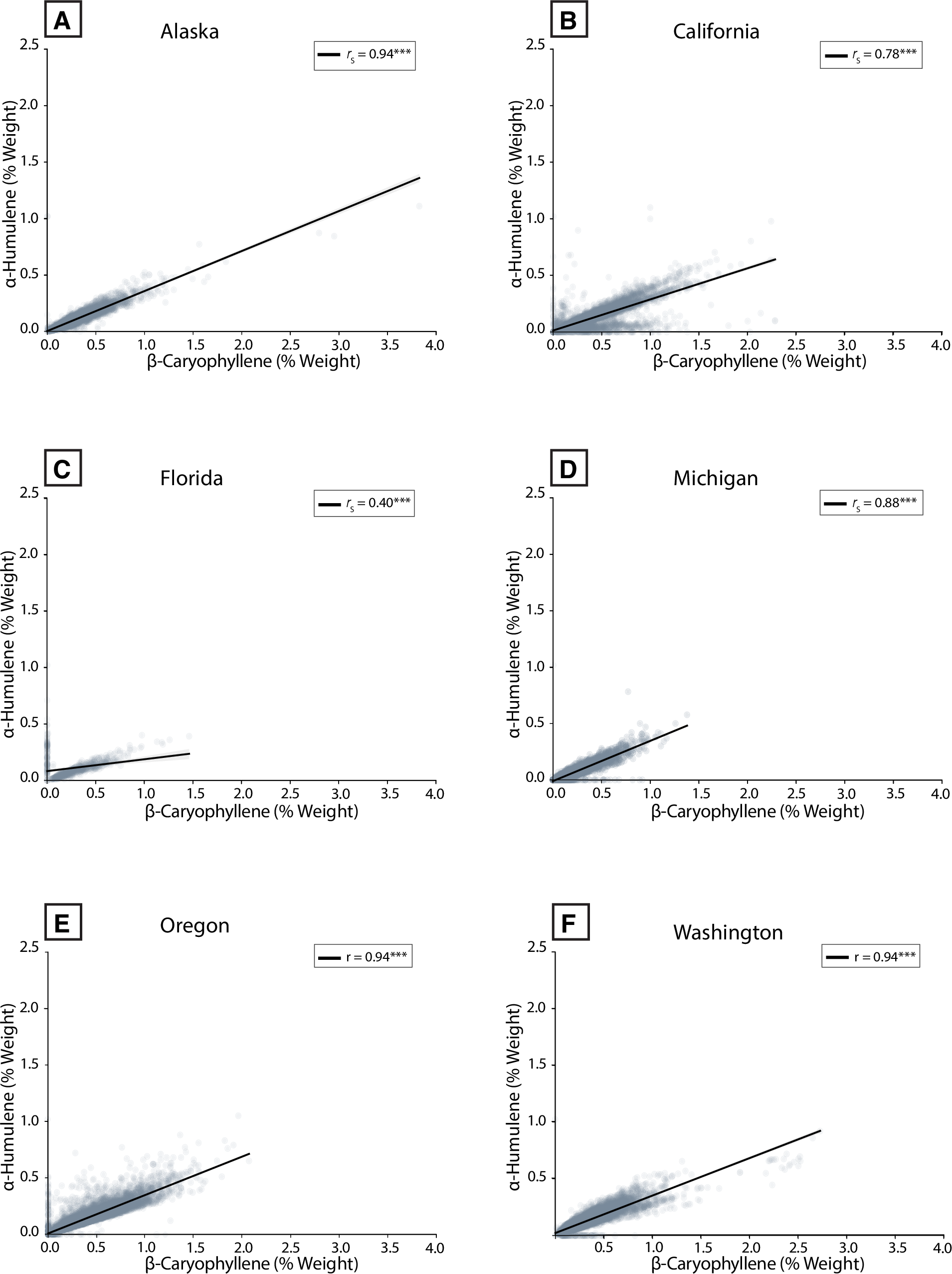

Supplement: S4 Fig — ***P < 0.0001. (TIF) [file pone.0267498.s004.tif]

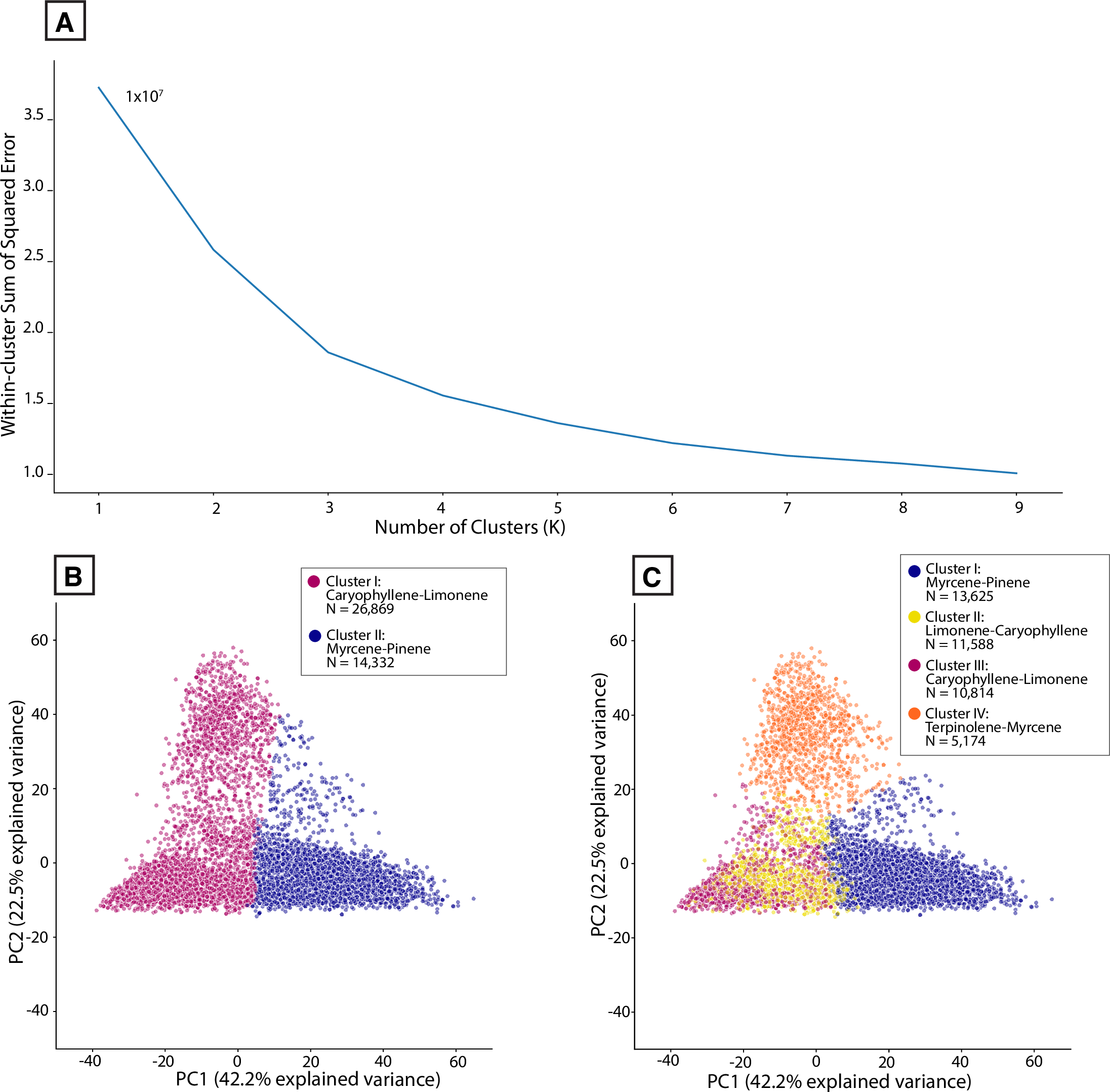

Supplement: S5 Fig — (A) Line plot showing the relationship between number of clusters in k-means clustering and within-cluster sum of squared errors, using THC-dominant sample terpene data. “Elbow point” was determined to be at k = 3. (B) PCA scores for all THC-dominant samples plotted along PC1 and PC2, color-coded by k-means cluster labels, k = 2. (C) PCA scores for all THC-dominant samples plotted along PC1 and PC2, color-coded by k-means cluster labels, k = 4. (TIF) [file pone.0267498.s005.tif]

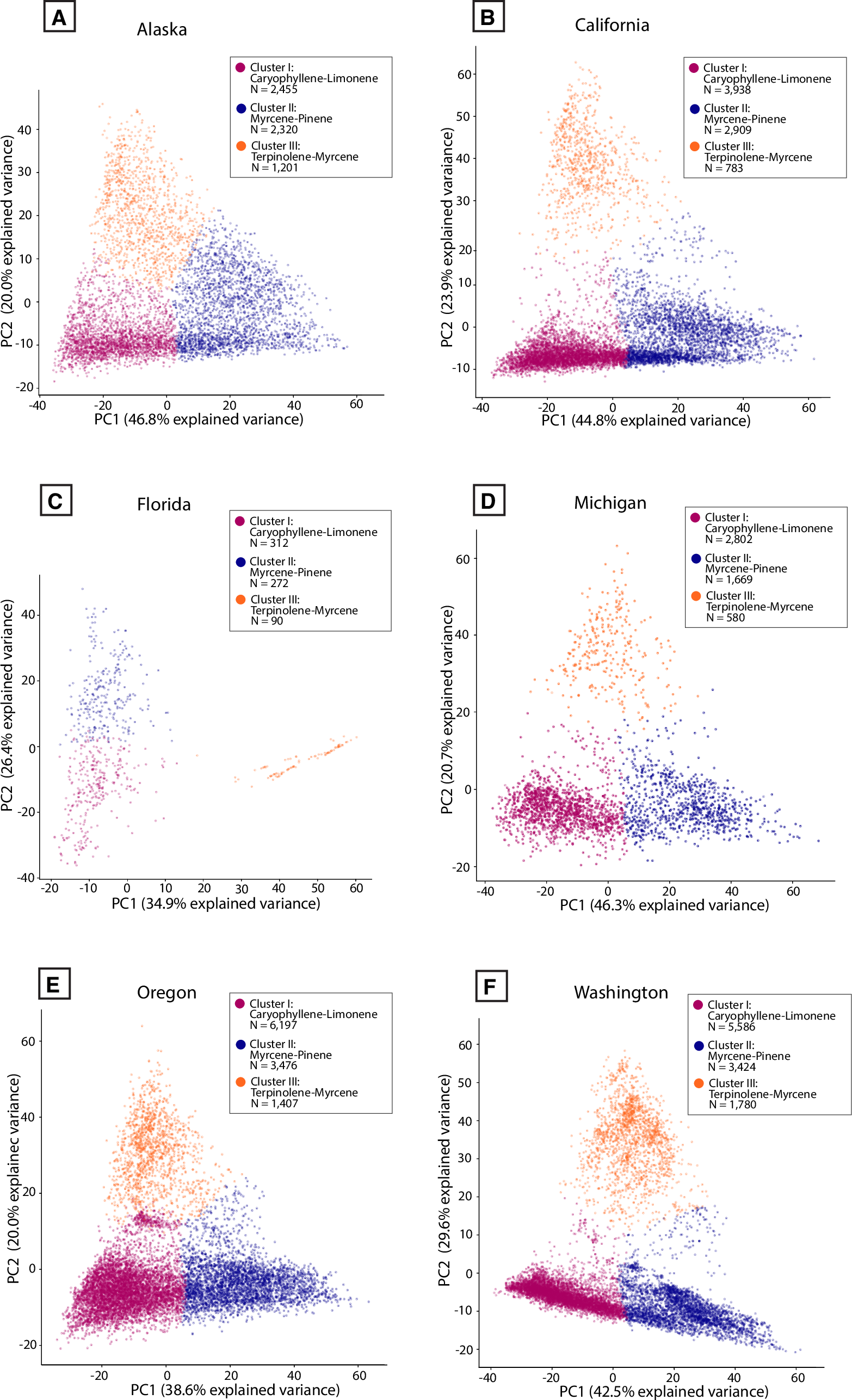

Supplement: S6 Fig — (TIF) [file pone.0267498.s006.tif]
