# Supplementary material for: The phytochemical diversity of commercial Cannabis in the United States
Source: PLoS One. 2022 May 19;17(5):e0267498. doi: 10.1371/journal.pone.0267498 (PMC9119530; doi:10.1371/journal.pone.0267498)
Supplement: Attachment — Submitted filename: PLOSONE_AppealRequestForm_filled.pdf [file pone.0267498.s007.pdf]

PLOS ONE Appeal Request Form

Please fill out the below form with the relevant information. We require a response to all three sections before we can evaluate your appeal request.

---

1. Academic Editor

Our standard process is to consult Academic Editors on appeals on decisions they handled. If you have any concerns that a consultation with the Academic Editor may unduly affect the evaluation of your appeal, please indicate so along with a brief summary of the reasons for your concerns.

☐ I do not have any concerns about the Academic Editor being consulted on this appeal.

X☐ I do have concerns that the Academic Editor being consulted may unduly affect the evaluation of my appeal for the following reasons:

If the academic editor was unaware that submitting manuscripts to well-known preprint servers is standard practice in science and explicitly encouraged by PLOS, then I am not sure they are paying sufficient attention to their duties as an editor.

2. Editorial Policies

If you have concerns that the decision deviates from one of our editorial policies, please provide us with some brief notes on the specific policy.

☐ I do not have any concerns about this decision deviating from PLOS ONE's editorial policies.

X☐ I do have concerns that this decision deviated from PLOS ONE's editorial policies for the following reasons:

Any clear deviation from editorial policies is a concern. This has taken many weeks and hours of work to resolve. I have carefully documented what has happened, how much time it has taken, and that editorially policies were contradicted by the journal.

3. Response to Reviewers:

Please provide a point-by-point response to the reviewers and any Academic Editor's comments in a separate document.

X☐ I confirm that I have attached a Response to Reviewers as a separate file to this email in addition to this form.

There is nothing to respond to beyond noting that it was inappropriate to reject our paper for being posted on a preprint server.

4. Track Changes Manuscript:

If you made any updates to your manuscript, please also provide a 'Track Changes' version of your manuscript that shows any changes you may have made to your submission.

X☐ I confirm that I have attached a 'Track Changes' version of my MS as a separate file to this email in addition to this form and Response to Reviewers.
