# Supplementary material for: The phytochemical diversity of commercial Cannabis in the United States
Source: PLoS One. 2022 May 19;17(5):e0267498. doi: 10.1371/journal.pone.0267498 (PMC9119530; doi:10.1371/journal.pone.0267498)
Supplement: Attachment — Submitted filename: Answers for Reviewers.docx [file pone.0267498.s008.docx]

**Reviewer #1: 1. Several issues concerning the material and method section are of concern:**

A. ANALYTICAL PROCEDURES: The material and methods section should be revised to include a section about analytical procedures. Detailed analytical procedures of the chemical analyses procedures must be included as is acceptable for scientific papers. The very general description given so far mainly for instrumentations is insufficient.

Thank you for your suggestion. We reworded the Methods section and included within our Github repository a more detailed summary of the methods used by each lab (lines 985-986). We asked each testing lab to submit to us a summary of their analytical procedures, including information about their sample selection, preparation and analysis. Because there is approximately one full page of information from each of the six labs, we included these as attachments within our Github repository. We also included other updates in the Data Collection section of Methods to do with the source material (lines 890-893), and a table with sample information.

B. Results from several laboratories using unknown analytical procedures of varied instrumentations were pulled for the study. This is a very large disadvantage, since large variations in chemical analysis results for cannabinoids and terpenes are known to results from analysis at various laboratories; and no standardization was conducted in the present study. It can therefore not be excluded that variabilities reported in the study, for example regional variability, do not source from variations in accuracy of the analytical techniques. For this affect, the detailed analytical procedures used in each laboratory should be detailed in the manuscript (as is mentioned in point A above). Furthermore, the authors should consider to use reference material to be analyzed by all laboratories, and to normalize the data used for the study based on identified inconsistencies between laboratories, prior to the data evaluation conducted in the study.

Although we agree that adding more detail about analytical procedures improves the paper (see above), we disagree that having multiple laboratories is a disadvantage, at least in certain respects. Our study analyzes real-world data reflecting the measurements attached to *Cannabis* used by consumers in various legal markets and analyzes how well these match up with commercially used product labels. In addition, we identified patterns of chemical variation that are consistent across these different labs. Our analysis shows that despite any methodological differences between labs, we still observe many of the same basic patterns in cannabinoid and terpene variation across regions (e.g. Figures S2, S3, S4, S6). This is related to why we confirmed that certain correlation patterns among specific terpene pairs (Figure 3) held across labs, as some of these have a known biological origin and have been previously reported. Thus, there are patterns of phytochemical variation that are robust to any methodological differences between labs.

There may be further, finer-scale patterns of variation that are not apparent in all regions (e.g. Figure S6, seen as fine-scale differences in the PCA plots). As the reviewer points out, we are *not* able to discern whether any observed regional differences (e.g. differences in mean total THC concentration) reflect true sample differences, methodological differences, or both.

To make all of this more clear, we have changed text at lines (e.g. lines 274-276) to make it more clear to readers that we cannot draw firm conclusions about whether differences observed across regions reflect true sample variation and/or methodological differences, and that certain patterns observed across all labs are consistent with previous observations in the literature.

C. THE SOURCE OF THE SAMPLED MATERIAL: A proper description of the source of the material used for the study is required. Are these samples from dispensaries of marketed products? From growers? From consumers? Etc. I suppose that these are results of the commercial labs datasets. More detailed information about the source of the samples should be supplied.

This is an important point and we agree that this was not made clear enough in the original submission. We added a better description of the source of the material in the methods section (lines 890-893) and Introduction (lines 274-276) to make it more clear that the source material we analyzed is submitted by cultivators in order to fulfill legal testing requirements before being sent to retail locations.

2. Another problematic issue that should be addressed is that some text parts reads like a commercial/advertising in a form that is not suitable for scientific publications. Specifically: A. The first paragraph of the results section: You can mention the source of the data ones in the manuscript, and all other text about Leafly (this also includes line 660-662 ) and all other mentions of leafy in the manuscript such as "leafy supplied.., leafy included… " should be removed.

We appreciate the suggestion and removed these lines to avoid redundant mentioning of data sources. We mention Leafly as the source once up front (at the end of Introduction), and again in the Methods section. Other mentions have been removed.

B. Line 704-723: This section and the details of the lab are of commercial nature, and have no place in a M&M section of a scientific report. It should be removed from the manuscript. Relevant information pertaining to the M&M can be added to the text. Some credit, albeit not in the form it is now written can be added to the acknowledgment section.

Thank you, we modified this section, added a repository link containing several pages of detail pertaining to laboratory methods, and updated the acknowledgements section.

3. Scientific background: The introduction lacks a discussion about potential reasons other then genetics diversity between strains for the variability in the secondary metabolite profile between samples. There is a growing body of scientific information about effects of cultivation conditions such as mineral nutrition (N, P, K, ), light, and plant architecture on secondary metabolite production in cannabis.

Thank you for pointing this out, we added sentences (lines 193-200) in the introduction addressing the possible environmental effects on secondary metabolite production, in addition to the portions of the Discussion section where we discussed environmental factors (e.g. Discussion paragraph two).

4. Terminology: The use of the word cannabis in the manuscript to describe cannabis derived products as well as cannabis plant material products and the cannabis plant is misleading and confusing. It is confused with concentration in the cannabis plant. Therefore them manuscript should be revised throughout for terminology, to distinct between cannabis plant material, and cannabis derived products. One example out of many for this issue is line 101 – it is unclear whether this sentence refers to differences in cultivation in one area, or products distributed in one area.

Thank you for the suggestion, we went through the manuscript and made sure that the wording was more consistent. We now distinguish between *Cannabis* (the plant), *Cannabis*-derived flower products (the data we analyzed), and other derivative products.

5. Abstract: remove the words "After careful descriptive" from line 32. Just describe your results and conclusions. All scientific work should be carefully conducted.

These words have been removed.

6. Line 35-36. "However, certain labels are statistically overrepresented for specific chemotypes" - This is not clear.

We have updated the language here.

7. The last sentence of the abstract: it is not clear from the abstract why this is the conclusion of the study.

We have updated the language here.

8. Line 52-53: remove the uncommon names, and stay focused in the introduction on the topic of study.

We have updated the language here.

9. The sentence in lines 54-56 is not supported by references.

We have opted to remove this sentence as it is largely redundant with the next sentence (supported by citations 11-12).

10. Throughout the manuscript, Δ-9-tetrahydrocannabinolic should be corrected for the proper writing.

Thank you. We made sure we corrected any misspellings in the compound name throughout the manuscript.

11. Paragraph starting in line 70: this paragraph is written in a popular style of writing. It needs to be revised to reflect scientific writing style for chemical pathways and processes.

We made substantial changes to this paragraph to reflect the scientific writing.

12. The paragraph in lines 119-134 is to long. The discussion concerning medical effect of the metabolites interactions should be reduced considerably.

We have consolidated this paragraph into fewer sentences.

13. The lists in pages 32-33: should be converted to regular text in sentences (not lists).

This has been done.

14. I am not an expert of the statistical methods used and the curve fitting techniques used. But judging the appearance of the regression lines in figure 2, it seems that for many of the sub-figures that regression lines are extrapolations and does not visually fit the trend for the bulk majority of the data. These curve fits should be reevaluated.

We have re-done our regression analysis and visualizations for Figure 2. While there was nothing technically wrong about the regression analysis we performed, the regression lines did not visually fit the data as one might expect due to the presence of outliers within each panel relative to the bulk of data points. We re-ran the regression analysis on each sub-figure, excluding extreme outliers. These were defined using the median absolute deviation method with a conservative threshold of 3.5 standard deviations on both x and y values, for each chemotype. This is also explained in the Methods section. We have updated Figure 2 accordingly.

15. Figure 3: because a uniform scale was selected for the Y axis for all terpenes, it is impossible to see the trends for most (minor) terpenes.

Our intention here was to show the relative abundance across most of the major terpenes, in order to make it clear that some terpenes are much more abundant than others for most samples.

We could make an additional supplemental plot with different y-axes for each terpene, but such a figure would likely span multiple panels or even multiple supplemental figures. We are happy to add this if the reviewer thinks it is critical for the paper, although we already have a large number of figures. As more and more compounds get tested over time, and more total data is accumulated, we think a finer-scale analysis of all of these minor compounds could be the subject of a separate study.

**Reviewer #2: PONE-D-21-34155**

The phytochemical diversity of commercial *Cannabis* in the United States

Smith et al.

The manuscript The phytochemical diversity of commercial Cannabis in the United States reports the results of an analysis of aggregated cannabinoid and terpene plant secondary metabolite data in the species Cannabis sativa. The authors interpret this data thoughtfully and provide new illumination on several standing topics. Additionally, the authors propose a helpful model to help explain the chemotaxonomic chemical relationships that help explain patterns seen within this dataset.

Overall, this work is compelling and adds tangible value to the larger discussion of several critical topics in Cannabis research. For the most part, the statistical approach is strong but requires additional scrutiny before publication. The authors should be praised for making both the underlying dataset and python code easily accessible online.

The introduction of seems to lack an understanding of the underlying genetic pathways that control biosynthesis of cannabinoids and terpenes in cannabis. This is important information to interpret their findings and is largely neglected. A more comprehensive literature review of cannabis genetics and biosynthetic pathways would greatly strengthen the value of this work.

I recommend that this paper undergo major revisions before resubmission.

Thank you very much for the kind words and for taking the time to review our manuscript. We address your questions below. In addition to the paragraphs we already have discussing these issues (e.g. paragraphs 3-4 of the Introduction), we have added further elaborations and citations to discuss the genetic pathways and biosynthesis pathways that govern cannabinoids & terpene production. These amendments to the Introduction (lines 83-88) complement what we already included in the Discussion section (paragraph 4).

There are several specific elements that if improved would make this work considerable stronger.

1. The terminology used throughout this paper often feels muddled or inconsistent. For example, the terms “sample”, “strain”, “variety” are not well defined. To unify cannabis research with the larger field of plant science, this paper reinforces the meaningless term "strain" as valid nomenclature for cannabis cultivars. This usage is unhelpful and should be deprecated. There are more precise and specific terms e.g., cultivar, population, selection, hybrid, landrace that would bring more clarity to this work and not muddle scientific research.

We have updated this terminology throughout the manuscript. In particular, we make more frequent use of the term “cultivar” and only use “strain name” in quotes to help emphasize that we are evaluating samples as they are labeled in the commercial industry, and are not endorsing this as the preferred nomenclature. See also the Introduction and Discussion for further explanation of how labels are used in commercial settings vs. by scientists.

2. The lack of discussion and analysis of sample duplication or potential duplications is troubling. The manuscript claims that the study uses 90 thousand samples is misleading. In fact, the paper uses repeated measurements of only 3,439 unique named cultivars but does not state this. There should be much more attention paid to statements such as “The final number of samples was 89,923” which easily misleads the casual reader to think that this study is capturing 25 times more cultivars than it is. For example, there are over 1000 “samples” of ‘blue-dream’, ‘original-glue’, ‘dutch-treat’, and ‘gsc’ each. In the raw dataset, 481 cultivars have only one observation. This is inconsistency is not addressed and should be taken very seriously. Please report the number of cultivars and measures of dispersion in the abstract and introduction.

This is a good point. We have added counts of unique cultivators and unique cultivar names found in each lab dataset, before and after cleaning, as well as the total number of unique cultivar names found in the dataset, which was 3,087 cultivars after data cleaning.

3. The authors of this manuscript do not seem to fully grasp that the mechanism of THC:CBD ratio is well-defined from a genetics perspective, e.g. the genotype at the B locus encodes THCA synthase or CBDA synthase. The term “Balanced THC:CBD” simply reflects a heterozygous individual at this locus. Please see the work of de Meijer and others who have clearly defined this locus (<https://doi.org/10.1093/genetics/163.1.335> or <https://genome.cshlp.org/content/29/1/146.short>). Additionally, the authors should consider carefully how to use the term “chemotype”. In most cannabis research, the term “chemotype” is used in a discrete and specific way (e.g., “Chemotypes I through V”), although in this paper, the authors tend to switch back and forth between using this term in an informal way to discuss any secondary metabolites. This will be confusing to many readers.

Thank you for pointing out the use of the word ‘Chemotype’ throughout the manuscript. We have followed previous literature on this point. “Chemotype I-III” has been used at least since Hillig in the 1990s to refer to cultivars based on their THC:CBD ratio. This ratio, as the reviewer points out, is a reflection of underlying biochemical differences between cultivars with different genotypes. Because only certain readers will be familiar with the Chemotype I-III nomenclature, we additionally included the labels, “THC-dominant,” “Balanced THC:CBD”, and “CBD-dominant” to reduce cognitive load on readers who have not memorized the other nomenclature. Indeed, for a previously published paper (Jikomes & Zoorob, 2018), the terms “THC-dominant,” “Balanced THC:CBD”, and “CBD-dominant” were added as a result of the peer review process. We understand that this descriptive wording illustrates the phenotype but not the genotype related to cannabinoid production.

We have amended and expanded the discussion of the genomic basis for both cannabinoid and terpene variation, which you will see in both sections. Because the term “chemotype” simply refers to any clearly identifiable aspect of chemical phenotype, it is not necessarily limited to THC:CBD ratios. We have amended our wording throughout the manuscript to help make it more clear when we are referring to a THC:CBD chemotype vs. a terpene profile chemotype defined by cluster analysis.

We added explanation on the genes related to cannabinoid production in the introduction (lines 83-87), which was initially thought to be a one-locus-two-allele type of model, but now we know it’s multiple genes in close proximity.

4. The authors often replace missing data with zeros (e.g., line 854). I am not at all confident that this is the appropriate statistical treatment.

This was done in cases where null values were present, which represent instances where a compound was either absent or, if present, present below the Limit of Quantification. As such, it is appropriate to express these values as zeros. An alternative approach which can be done is to set the values equal to the Limit of Quantification for that compound.

We reran our PCA analysis on cannabinoids (Fig 1D) and terpenes (Fig 5B-D) where we filled missing data with the largest LLOQ we had for that compound (i.e, 0.1 for cannabinoids and 0.01 for terpenes). The PCA results were not significantly impacted; this is evidenced by the minor differences in percentage of variance explained for the first component. For Fig 1D, we saw only a 0.2% difference on PC1 variance explained when using this alternative approach, and Fig 5B we saw no difference in PC1 variance. We have added this fact to the Methods section.

5. The authors do not correctly state the legal definition of hemp as total THC in line 83. See 2021 Final Rule <https://www.federalregister.gov/documents/2021/01/19/2021-00967/establishment-of-a-domestic-hemp-production-program>.

Thank you. We amended lines 159-161 to quote directly from the .gov source above.

6. In line 83, the authors mention that the 0.3% threshold was “arbitrarily defined”. This is not precise; the threshold was largely based on previous work by Ernest Small (see <https://www.jstor.org/stable/4253607>) who thought at the time that the upper limit of THC in hemp would always be less that 0.3%.

Thank you. We amended this description of the THC threshold for hemp.

7. The authors should consider including the biochemical pathways of cannabinoid and terpene biosynthesis and their molecular structures. Proving a prior on how these metabolites are synthesized would be an illuminating compliment. For example, a clearer model of cannabinoid biosynthesis would help clarify that the variation observed in this data is likely a result of several severe genetic bottlenecks / founder effects / selective sweeps within the larger pool of cannabis germplasm, rather than random variation that might be observed within natural populations.

Thank you. We added citations for the biochemical pathways on lines 83-87 in the Introduction. We find it unnecessary to include figures of the biochemical routes as the cannabinoid pathway has been clearly described, and the terpene pathways are still mainly unknown (Zager et al. 2019, Booth and Bohlman 2019, Both et al. 2017 and 2020, Livinsgton et al. 2020). We worry that adding chemical structures to the figure panels will create additional clutter, but have emphasized in the text other literature where readers can obtain this structural information.

8. Figure 10 is very useful and presents a novel model to help explain metabolic variation in cannabis germplasm. The authors might consider using a more quantitative visualization of this data, rather than representing relationships with line width. For example, a Sankey diagram might be more appropriate to show this relationship more quantitatively (see <https://en.wikipedia.org/wiki/Sankey_diagram>).

This is an interesting suggestion. We considered some alternative plots for this figure, but opted for a qualitative depiction for two reasons: (1) We already have multiple figures which display the main patterns of variation in a quantitative fashion, and (2) we wanted to provide an intuitive, qualitative model given that not all readers may be familiar with quantitative visualization methods such as PCA, UMAP, and others. We also obtained some feedback from colleagues that reinforced this choice.

9. In line 577, the authors state that “Marketing emphasizing Indica-labelled products as sedating and Sativa-labelled products as energizing are not borne out by our analysis of the underlying chemistry”. I personally believe that the authors assumptions are correct, but I am not confident that they can make this claim given the evidence that they present.

We have updated the language here to be more precise and appropriately conservative.

10. The authors gloss over that there appears to be no representation of hemp within this study. Some clarification or connection to work that has evaluated hemp secondary metabolites would be helpful.

Thank you for pointing that, however, given that hemp is not usually found in dispensaries, and our study is a description of the *Cannabis* sold legally in dispensaries, we do not include hemp for fiber or oil, but there are high CBD marijuana strains in our study. We have updated the text to make this more clear.

11. The authors exclude 0% cannabinoids from their analysis. This is probably correct, although cannabinoid knockout material does exist.

This is correct, but is very unlikely to be present in samples tested for the consumer market. Indeed, an extremely small number of samples (n=64) in the combined dataset had <1% cannabinoids. None of our statistical findings are impacted by inclusion of these.

12. The correlations and trendlines in Figure 2 look very strange, especially the “Balanced” comparison between total THC and total CBD. Please verify that this analysis is correct and appropriate.

Yes, this was a good catch. There was nothing technically wrong about the regression analysis we performed, the regression lines did not visually fit the data as one might expect due to the presence of outliers within each panel relative to the bulk of data points. We re-ran the regression analysis on each sub-figure, excluding outliers. These were defined using the median absolute deviation method with a conservative threshold of 3.5 standard deviations on both x and y values, for each chemotype. This is also explained in the Methods section. We have updated Figure 2 accordingly.

**Reviewer #3**: I found the paper to be well written and constructed. The intro could perhaps benefit from a reduction in length. The results section also at times blends commentary that could be moved to the discussion or methods sections. The discussion section really ties together the major findings of the analyses and highlights the importance of the work.

Thank you for these comments. We have attempted to reduce the length of the introduction, although we added text about the genetics and biochemistry of metabolite production at the suggestion of another reviewer.

I think an important analytical consideration to discuss is the magnitude of the significant differences for the groups, especially regarding the terpenes. While the clustering analyses are compelling, what are these significant differences really telling us about the samples? The terpenes are measured at a few percent at most, so how big are the significant differences between the groups in terms of terpene levels? I would also like to read some additional discussion on any outliers that stand out.

We investigated the differences in overall terpene levels between the k-means clusters. While the p-values for each group comparison were significant, the d-prime values ranged from 0.14 to 0.36, therefore the differences in total terpene levels between clusters is not very big.

The suggestion about outliers is interesting. We have added to the Discussion some commentary about some notable outliers we see in this dataset. There are other terpenes which are occasionally dominant. For example, ocimene is the dominant terpene in 1% of THC-dominant samples. Ocimene-dominant samples are found most commonly in both cluster A and C. Meanwhile, linalool is the dominant terpene in 0.7% of THC-dominant samples and is almost entirely associated with cluster A.

I recommend not using the term legal cannabis industry. Most state laws use the term marijuana, despite the historical baggage of that term. Either way, I think it should be clarified what is known about these the origins of these samples and how sample selections may have affected the results. While the methods describe the relationship between the testing labs the Leafly to some extent, I don't see anything about the lab / cultivator relationship--not everyone knows about the state testing requirements etc. I assume these are state licensed marijuana cultivators' samples. This would exclude hemp industry samples, many of which are genetically very similar to MJ (Grassa et al. 2021).

We have added commentary about sample origins to the Introduction and Methods (see above comments), updated the Methods to include the number of unique cultivators per region (see updated tables in Methods), and updated our use of ‘legal cannabis industry.’

Line number comments:

71: Varieties with > 15% CBGA are common in the US now. Same for THCV in some areas.

While such cultivars do exist, they are not yet common among consumer products and not represented in our dataset. This is mentioned at a couple places, e.g. lines 744-746.

89: Include de Miejer breeding papers and terms?

Yes, we have cited de Meijer (2003).

275 – 286: These analyses reflect highly unbalanced sample numbers in the dataset for type I, II, and III plants.

While this is true, we obtain the same result by iteratively conducting our analysis on random subsamples from each group, such that sample numbers are balanced. We have clarified this in lines 391-392

We performed a bootstrap simulation, sampling each chemotype group to be 245 products, then examined the average d-prime values from the bootstrapped distribution for each chemotype pair. We found that the bootstrapped average d-prime value for each pair was within 0.01 of the d-prime values reported on Figure 5E.
